# Osimertinib Plasma Trough Concentration in Relation to Brain Metastases Development in Patients With Advanced *EGFR*-Mutated NSCLC

**DOI:** 10.1016/j.jtocrr.2024.100656

**Published:** 2024-02-20

**Authors:** Judith L. Gulikers, G.D. Marijn Veerman, Merel Jebbink, Paul D. Kruithof, Christi M.J. Steendam, René J. Boosman, Ron H.J. Mathijssen, Vivianne C.G. Tjan-Heijnen, Johanna H.M. Driessen, Safiye Dursun, Egbert F. Smit, Anne-Marie C. Dingemans, Robin M.J.M. van Geel, Sander Croes, Lizza E.L. Hendriks

**Affiliations:** a Department of Clinical Pharmacy & Toxicology, Maastricht University Medical Centre+, Maastricht, The Netherlands; b CARIM School for Cardiovascular Disease, Maastricht University, Maastricht, The Netherlands; c Department of Medical Oncology, Erasmus MC Cancer Institute, Rotterdam, The Netherlands; d Department of Pulmonary Medicine, Erasmus Medical Center, Rotterdam, The Netherlands; e Department of Thoracic Oncology, The Netherlands Cancer Institute, Amsterdam, The Netherlands; f Department of Pulmonary Medicine, Catharina Hospital, Eindhoven, The Netherlands; g Department of Pharmacy and Pharmacology, The Netherlands Cancer Institute, Amsterdam, The Netherlands; h Division Medical Oncology, GROW – School for Oncology and Reproduction, Maastricht University Medical Centre+, Maastricht, The Netherlands; i NUTRIM School for Nutrition and Translational Research in Metabolism, Maastricht University Medical Centre, Maastricht, The Netherlands; j Department of Pulmonary Diseases, GROW – School for Oncology and Reproduction, Maastricht University Medical Centre+, Maastricht, The Netherlands; k Department of Pulmonary Diseases, LUMC, Leiden, The Netherlands

**Keywords:** NSCLC, Osimertinib, Brain metastasis, Plasma trough concentration

## Abstract

**Introduction:**

Brain metastases (BM) are common in patients with advanced *EGFR*-mutated (*EGFR*m+) NSCLC. Despite good BM-related outcomes of osimertinib, several patients still experience intracranial progression. A possible explanation is pharmacologic failure due to low plasma trough levels (C_min,SS_) and consequently limited intracranial osimertinib exposure. We investigated the relation between osimertinib C_min,SS_ and BM development or progression.

**Methods:**

A prospective multicenter cohort study, including patients receiving osimertinib for advanced *EGFR*m*+* NSCLC. At osimertinib start, patients were allocated to the BM or no or unknown BM cohort and were further divided into subgroups based on osimertinib C_min,SS_ (low, middle, and high exposure). Cumulative incidence of BM progression or development and overall survival were determined for each group.

**Results:**

A total of 173 patients were included, with 49 (28.3%) had baseline BM. Of these patients, 36.7% experienced BM progression, of which 16.7% in the low (<159.3 ng/mL), 40.0% in the middle, and 47.1% in the high (>270.7 ng/mL) C_min,SS_ subgroups. After 12 months, the cumulative incidence of BM progression for the BM cohort was 20% (95% confidence interval [CI] 2.6–49.0), 31% (95% CI:10.6–53.9), and 31% (95% CI:10.8–54.5) per C_min,SS_ subgroup, respectively. After 20 months, this was 20% (95% CI:2.6–49.0), 52% (95% CI:23.8–74.2), and 57% (95% CI:24.9–79.7), respectively. For the no or unknown BM cohort, 4.0% developed BM without differences within C_min,SS_ subgroups.

**Conclusions:**

No relation was found between osimertinib C_min,SS_ and BM development or progression in patients with advanced *EGFR*m*+* NSCLC. This suggests that systemic osimertinib exposure is not a surrogate marker for BM development or progression.

## Introduction

Brain metastases (BM) are diagnosed in approximately 25% of all patients with a first diagnosis of metastatic EGFR-mutated (*EGFR*m*+*) NSCLC, and this can rise to more than 50% during the course of the disease.^1^, ^2^, ^3^ Symptomatic BM are associated with a decrease in quality of life, and patients with BM have a worse prognosis compared with those without.^4^ The percentage of patients diagnosed with having asymptomatic BM is increasing as baseline screening for (asymptomatic) BM in stages II to IV NSCLC is advised in the European Society of Medical Oncology and the National Comprehensive Cancer Network guidelines.^5^, ^6^, ^7^

Standard first-line therapy for patients with metastatic *EGFR*m+ NSCLC is an EGFR tyrosine kinase inhibitor (TKI).^6^^,^^8^ Osimertinib, a third-generation EGFR TKI, has superior blood-brain barrier (BBB) penetration compared with first- and second-generation EGFR TKIs,^9^, ^10^, ^11^ resulting in improved central nervous system (CNS)-related outcomes.^12^, ^13^, ^14^ This, together with improved overall survival (OS) compared with first-generation TKIs and a favorable toxicity profile, led to osimertinib becoming the preferred first-line therapy.^6^^,^^8^^,^^14^

Despite better CNS efficacy of osimertinib, CNS progression occurs in 21% and 24% of patients treated with osimertinib in first line or in second line on acquiring the p.T790M resistance mutation, respectively.^14^^,^^15^ Possible reasons could be pharmacologic failure of osimertinib due to limited intracranial exposure, resulting in suboptimal CNS levels, or due to the development of molecular resistance mechanisms. The relevance of steady-state osimertinib plasma trough levels (C_min,SS_) in relation to CNS progression was evaluated in the OCEAN study enrolling patients with *EGFR*m+ NSCLC and radiotherapy-naive CNS metastases. No differences were found between patients with a low (<568 nM [284 ng/mL]) versus those with a high (≥568 nM [284 ng/mL]) osimertinib C_min,SS_, but it should be noted that the median osimertinib C_min,SS_ found in this Japanese cohort was substantially higher than what would be expected in a White population (approximately 200 ng/mL as reported by Rodier et al.).^16^, ^17^, ^18^

If low osimertinib C_min,SS_ is associated with a shorter time to brain parenchymal progression, this could be used as a potential surrogate marker of intracranial exposure with therapeutic implications (e.g., osimertinib dose escalation). As there is a lack of data regarding osimertinib C_min,SS_ measurements and BM-related outcomes, especially in White patients, we investigated the relation between osimertinib plasma C_min,SS_ and the development and progression of BM in patients with *EGFR*m*+* NSCLC treated with osimertinib.

## Materials and Methods

### Patients

A prospective multicenter cohort study was performed including patients from three tertiary Dutch hospitals, receiving osimertinib 80 mg once daily as treatment for advanced *EGFR*m*+* NSCLC between January 2015 and December 2021 at Maastricht University Medical Centre + (MUMC+) (Maastricht), Netherlands Cancer Institute - Antoni van Leeuwenhoek Hospital (NKI/AvL) (Amsterdam), and Erasmus Medical Centre/Cancer institute (ErasmusMC) (Rotterdam). This study was approved in each center (MUMC+; 2019-1080, NKI/AvL; IRBd19-192 and IRBdm20-218, ErasmusMC; 16-643, START-TKI; NCT05221372). Samples from patients included in this study were previously evaluated for other research questions by Van Veelen et al.,^18^ Agema et al.,^19^ Boosman et al.,^20^ and Veerman et al..^21^ All patients signed informed consent. Patients who underwent a dose reduction of osimertinib during treatment were excluded from the analyses, because the found plasma C_min,SS_ would not be representative for the total time of osimertinib treatment. Patients without an eligible osimertinib plasma trough concentration (C_min,SS_) sample, as specified subsequently, were also excluded from the analysis. Patient characteristics were collected from the medical files and included demographics, type of *EGFR* mutation, presence of parenchymal BM, presence of leptomeningeal metastasis (LM), previous treatments, osimertinib start date, line of osimertinib treatment, date and type of tumor progression on osimertinib (extracranial, brain, or both), and availability of brain imaging at start osimertinib.

The primary outcome for patients who already had BM at start osimertinib (BM cohort) was cumulative incidence of BM progression (BM-PD) in relation to the osimertinib C_min,SS_. For those who did not have known BM at start osimertinib (no/unknown BM cohort, i.e., those without brain imaging and no neurologic symptoms or with brain imaging revealing no BM), the primary outcome was cumulative incidence of BM development defined as a first registration of BM after start of osimertinib. The secondary outcome measure was median OS (mOS), defined as the duration from the index date (start of osimertinib) to the date of death due to any cause. Patients were censored if they were still alive at the last date of follow-up. The day of first prescription of osimertinib determined the index date, and patients were followed until they died, lost to follow-up, or last date of follow-up (December 31, 2021), whichever occurred first.

### Osimertinib Steady-State Trough Concentration

The osimertinib C_min,SS_ was determined in the plasma using previously validated liquid chromatography-tandem mass spectrometry assays,^22^, ^23^, ^24^ which were crossvalidated to some extent by sample exchanges between laboratories. Ideally, C_min,SS_ samples are taken 24 hours after the last osimertinib administration. Nevertheless, because these samples were drawn during regular hospital visits, the C_min,SS_ was estimated using a formula to correct for varying timing of sampling (Supplementary Appendix I).^25^

Osimertinib plasma samples were included in the analysis if (1) the plasma sample was taken within 6 to 36 hours after the last osimertinib intake, because the maximum plasma concentration is reached after approximately 6 hours; (2) the plasma sample was taken at least 15 days after start osimertinib (steady state); (3) blood withdrawal took place at least 3 months before progression, because an increase of the osimertinib plasma concentration was found shortly before progression.^18^ When multiple eligible samples per patient were available, the average C_min,SS_ of these samples was calculated and used in further analyses.

Patients in the BM and no/unknown BM cohorts were further allocated into subgroups based on osimertinib C_min,SS_ levels to distinguish between low and high osimertinib systemic exposure. We divided C_min,SS_ values into quartiles and used the 25th and 75th percentile as threshold values for low (C_min,SS,L_) (<159.3 ng/mL) and high (C_min,SS,H_) (>270.7 ng/mL) exposure, respectively. All remaining values were allocated to the middle exposure group (C_min,SS,M_).

### Diagnosis of BM and Progression

Development and progression of BM was assessed preferably on magnetic resonance imaging results or, if magnetic resonance imaging was contraindicated, on computed tomography scans. Scans were evaluated by the radiologist in regular care.

### Statistical Analysis

Baseline characteristics were descriptively stated and presented as the number and percentage for categorical characteristics. Numeric characteristics were presented as median and interquartile range (IQR). Development of BM in the no or unknown BM cohort and progression of BM was defined as the first diagnosis of BM according to brain imaging after start of osimertinib. Analysis of the cumulative incidence of progression and development of BM was performed using competing risk analysis, in which death and discontinuation of osimertinib treatment (due to extracranial progression or severe toxicity) were considered as competing risks. Cumulative incidence for BM progression and the development of BM was calculated using the cumulative incidence function, taking the competing events into account. The cumulative incidence was described at 6, 12, and 20 months after the index date. OS was defined as the time in months between start of osimertinib use and date of death. Patients alive at the last date of follow-up were censored. mOS, associated 95% confidence intervals (CIs), and hazard ratios (HRs) were calculated using the Kaplan-Meier method and Cox proportional hazard models. HR was adjusted for sex, line of treatment, *EGFR* mutation, and *TP53* status, as those were known to have an impact on osimertinib survival outcomes.^18^^,^^26^ The median follow-up time was calculated using the reversed Kaplan-Meier analysis. All statistical analyses were performed with the software package SAS version 9.4 (SAS Institute).

## Results

### Patients

**Figure 1:**
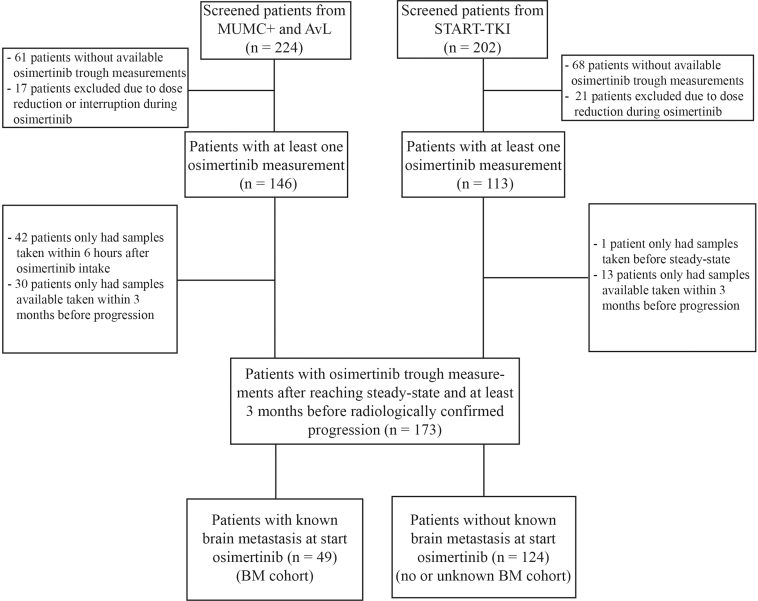
Flowchart inclusion and exclusion of patients. AvL, Antoni van Leeuwenhoek Hospital; BM, brain metastases; MUMC+, Maastricht University Medical Centre +; TKI, tyrosine kinase inhibitor.

Between January 2015 and December 2021, 442 patients started with osimertinib treatment for advanced *EGFR*m+ NSCLC (Fig. 1). In total, 173 patients were eligible, of which 49 (28.3%) were diagnosed with BM at the start of osimertinib treatment (BM cohort). Of these patients, 15 (30.6%) also had LM at start of osimertinib. Most patients were female, and this percentage was similar in both cohorts (71.4% BM cohort versus 68.5% no or unknown BM cohort; Table 1). Approximately half of the patients received osimertinib as second-line treatment (49.0% BM cohort and 50.8% no or unknown BM cohort), and the most prevalent prior TKI was erlotinib (51.5% BM cohort and 65.3% no or unknown BM cohort). An exon 19 deletion was the most common mutation (55.1% in the BM cohort and 61.3% in the no or unknown BM cohort). Baseline patient characteristics specified per subgroup are described in Supplementary Appendices II and III. The median osimertinib C_min,SS_ was 223.9 ng/mL (IQR 164-293) in the BM cohort and 210.0 ng/mL (IQR 156-256) in the no or unknown BM cohort (*p* = 0.71).

**Table 1:** Baseline Characteristics of Patients With and Without Known BM at Start Osimertinib

Characteristics		BM Cohort (n = 49)	No or Unknown BM Cohort (n = 124)
Female sex, n (%)		35 (71.4)	85 (68.5)
Age, median (IQR) in y		64 (58-68)	65 (58-73)
BMI, median (IQR) in kg/m^2^		24.1 (21.5-26.6)	24.5 (22.7-27.8)
Smoking status, n (%)	Never	22 (44.9)	72 (58.1)
	Current	9 (18.4)	4 (3.2)
	Former	18 (36.7)	47 (37.9)
	Unknown	0	1 (0.8)
TP53 mutation, n (%)		22 (44.9)	49 (39.5)
Diagnosis of leptomeningeal metastasis		15 (30.6)	NA
Line of treatment, n (%)	1	16 (32.7)	29 (23.4)
	2	24 (49.0)	63 (50.8)
	≥ 3	9 (18.4)	32 (25.8)
Prior cranial radiotherapy, n (%)	WBRT	7 (14.3)	NA
	SRT	8 (16.3)	NA
	No	34 (69.4)	NA
Prior TKI(s), n (%)	Erlotinib	17 (51.5)	65 (65.3)
	Afatinib	4 (12.1)	8 (9.5)
	Gefitinib	5 (15.2)	18 (16.8)
Mutation, n (%)	Exon19del	27 (55.1)	76 (61.3)
	L858R	13 (26.5)	33 (26.6)
	Other	6 (12.2)	11 (8.9)
	Exon19del + L858R	3 (6.1)	4 (3.2)

BM, brain metastases; BMI, body mass index; IQR, interquartile range; NA, not applicable; SRT, stereotactic radiotherapy; TKI, tyrosine kinase inhibitor; TP53, tumour tumor protein p53; WBRT, whole brain radiotherapy.

### Cumulative Incidence of Brain Metastases in Relation to Osimertinib C_min,SS_

**Figure 2:**
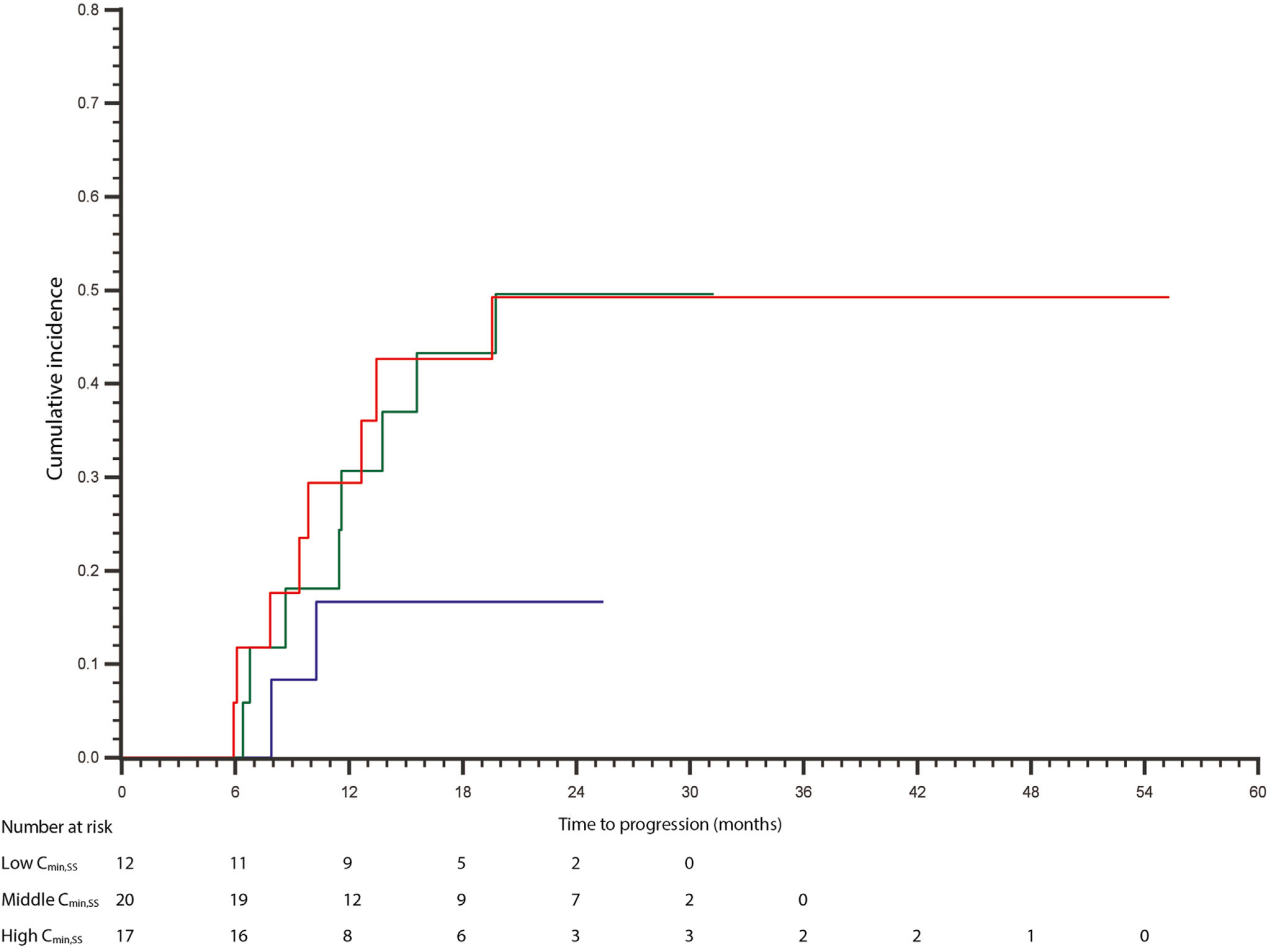
Cumulative incidence of BM progression in patients in the BM cohort. C_min,SS,L_ (<159.3 ng/mL) (blue); C_min,SS,M_ (159.3–270.7 ng/mL) (green); C_min,SS,H_ (>270.7 ng/mL) (red). BM, brain metastases; C_min,SS_, plasma trough levels.

The overall median follow-up was 22.8 months, 24.9 months (95% CI:3.2–35.8 mo) for the BM cohort and 21.4 months (95% CI:3.9–40.5 mo) for the no or unknown BM cohort. At data cutoff (December 31, 2021), 38.7% of the patients still used osimertinib, of which 19 patients (38.8%) in the BM cohort and 48 patients (38.7%) in the no or unknown BM cohort. Progression of BM under osimertinib or new BM was diagnosed in 18 of 49 patients (36.7%) in the BM cohort, of which two of 12 patients in the C_min,SS,L_ group (16.7%), eight of 20 patients in the C_min,SS,M_ group (40.0%), and eight of 17 patients in the C_min,SS,H_ group (47.1%). After 6 months, cumulative incidence was 0% for the C_min,SS,L_ and C_min,SS,M_ subgroup and 0.6% (95% CI:0.4%–25.5%) for the C_min,SS,H_ subgroup. After 12 months, the cumulative incidence was 20% (95% CI:2.6%–49.0%) in the C_min,SS,L_ subgroup, 30.8% (95% CI:10.6%–53.9%) in the C_min,SS,M_ subgroup, and 31.3% (95% CI:10.8%–54.5%) in the C_min,SS,H_ subgroup, respectively. This remained the same for the C_min,SS,L_ subgroup and increased to 51.9% (95% CI:23.8%–74.2%) in the C_min,SS,M_ subgroup and to 57.0% (95% CI:24.9%–79.7%) in the C_min,SS,H_ subgroup after 20 months. Time to BM progression was not significantly different compared with C_min,SS,M_ or C_min,SS,H_ (*p* = 0.289) (Fig. 2). Of the no or unknown BM cohort, five (4.0%) of 124 patients developed BM during use of osimertinib. Within the C_min,SS_ subgroups of the no or unknown BM cohort, a similar time to BM development was noticed (Supplementary Appendix IV).

### OS in Relation to Osimertinib C_min,SS_

In total, 53 of 173 patients (30.6%) died during the follow-up. mOS was 35.4 months (95% CI:27.1–not reached [NR]). In the BM cohort, 18 (36.7%) of 49 patients died, of which one in the C_min,SS,L_ group (8.3%), nine in the C_min,SS,M_ group (45.0%), and eight in the C_min,SS,H_ group (47.1%). For patients in this cohort, mOS was NR in the C_min,SS,L_ group, 27.3 months (95% CI:15.8–NR) in the C_min,SS,M_ group, and 26.2 months (95% CI:11.7–NR) in the C_min,SS,H_ group (Supplementary Appendix V). No differences in HRs were found when comparing patients in the C_min,SS,L_ subgroup and the C_min,SS,M_ subgroup (4.65 [95% CI; 0.50–43.10]) or when comparing patients with a C_min,SS,L_ subgroup with patients in the C_min,SS,H_ subgroup (4.19 [95% CI; 0.42–41.40]). For the no or unknown BM group, 35 (28.2%) of 124 patients died, of which eight (25.8%) in the C_min,SS,L_ group, 14 (20.9%) in the C_min,SS,M_ group, and 13 (50%) in the C_min,SS,H_ subgroup. The mOS was 35.4 months (95% CI:27.2–NR) in the C_min,SS,L_ subgroup, NR (95% CI:21.8–NR) in the C_min,SS,M_ subgroup, and 19.2 months (95% CI:15.8–NR) in the C_min,SS,H_ subgroup. The OS of the C_min,SS,L_ subgroup did not differ when comparing with the C_min,SS,M_ subgroup (HR 1.32 [95% CI 0.53–3.33]), but it was improved when compared with the C_min,SS,H_ subgroup (HR 4.47 [95% CI 1.64–12.19]) (Supplementary Appendix VI).

## Discussion

As data regarding osimertinib systemic exposure (i.e., plasma C_min,SS_ as a surrogate marker of AUC_0–24,SS_) in relation to intracranial progression are lacking in White patients, we evaluated whether there was an association between osimertinib C_min,SS_ and the development or progression of BM in patients with advanced *EGFR*m*+* NSCLC. Regardless of the presence of baseline BM, we found no correlation between C_min,SS_ and cumulative incidence of intracranial progression or metastasis development. Our results suggest that cerebral pharmacologic failure does not depend on the osimertinib plasma exposure, that is, a low C_min,SS_ is not the main driver of intracranial progression, and imply that other mechanisms play a role.

The number of studies investigating the effect of systemic osimertinib exposure on BM progression is scarce. In the OCEAN study, BM-PFS in relation to osimertinib plasma trough levels was evaluated in 37 Japanese patients with p.T790M+ NSCLC and, mainly asymptomatic, baseline CNS metastases. In line with the current study, the OCEAN study did not find significant differences in terms of BM-PFS between patients with an osimertinib plasma C_min,SS_ below (n = 18) or above (n = 19) the median of 284 ng/mL, with median BM-PFS of 12.7 versus 5.3 months (*p* = 0.357), respectively.^16^ Notably, the cutoff value of 284 ng/mL was substantially higher compared with our current data. Higher median osimertinib concentrations (median C_min,SS_ of 478 ng/mL) were also found in the APOLLO study (N = 12), evaluating the intracranial response in Chinese patients with p.T790M+ *EGFR*m+ NSCLC and CNS metastasis.^27^ Possibly, racial differences (e.g., CYP3A genotype or phenotype and body composition differences, and environmental factors) might play a role.

Patients included in the current study were previously described in other studies evaluating different research questions, but the specific question whether plasma levels are associated with BM outcomes was not evaluated.^18^, ^19^, ^20^, ^21^ We think that our study adds to the available literature. Furthermore, we did not use the proposed cutoff of C_min_ 166 ng/mL, because we found that a large proportion of our patients had a C_min_ above this value.

As the osimertinib C_min,SS_ values in our study varied from 85 ng/mL to 680 ng/mL, interpatient variability was substantial, and, consequently, large variations in intracranial exposure are also expected. Reasons for improved or reduced intracranial efficacy could potentially be clarified by differences in permeability of the BBB, leading to intracranial concentrations lower than needed to reach the IC90 or IC50 and therefore to be effective in most patients. De Leeuw et al.^28^ reported osimertinib CSF concentrations between 3.4 and 5.0 ng/mL in three patients with osimertinib 80 mg once daily in steady state. In these patients, the IC50 for tumor cells with an exon 19 deletion or exon 21 L858R mutation would have been reached, given the IC50 of approximately 4.0 ng/mL and 3.1 ng/mL, respectively. Nevertheless, these osimertinib CSF concentrations would not have been high enough to reach the IC90 for these mutations (250 ng/mL and 175 ng/mL, respectively).^29^ Another study evaluating osimertinib CSF concentrations reported a larger range of osimertinib CSF concentrations varying from 2.6 ng/mL to 15.1 ng/mL, but only four of 12 patients had complete or partial intracranial response, and this was still lower than the needed concentration to reach the IC50 or IC90.^27^

The FLAURA study, investigating osimertinib in first line, reported better PFS and OS for patients with an exon 19 deletion in comparison to patients with an exon 21 L858R point mutation.^26^ This difference in systemic PFS could also occur in BM-PFS, as was also reported by the OCEAN study.^16^ Nevertheless, a competing risk analysis was not performed, and it is unknown whether the censored cases in the OCEAN study were due to death owing to extracranial progression or whether the patient was still on osimertinib without progression at data cutoff. In the current study, exon 19 deletions had a higher prevalence in tumors of patients with a middle or high C_min,SS_ in the BM cohort (60.0% and 58.8%) compared with the prevalence in patients with a low C_min,SS_ (41.7%). The exon 21 L858R point mutation was present in more tumors of patients with a low C_min,SS_ in comparison to those with a middle or high C_min,SS_ (41.7% versus 25.0% and 17.6%, respectively) (Supplementary Appendix II). Although in theory this could have affected the results regarding time to BM-PD in this cohort, the number of patients experiencing BM-PD in the low C_min,SS_ subgroup was lower compared with those in the middle or high C_min,SS_ subgroups, despite the higher number of patients with an exon 21 L858R point mutation in the low C_min,SS_ subgroup. The number of patients included in each subgroup was too low to further divide the study population based on type of *EGFR* mutation.

Intracranial failure of osimertinib may also be due to differences in molecular profile of the intracranial and the primary tumor, either already at start of the treatment or developed in the course of the disease.^30^^,^^31^ In addition, a study by Adua et al.^32^ described molecular features of BM in mice after acquired resistance to osimertinib. They noted that EGFR activity in the tumor tissue of these mice was still decreased after resistance to osimertinib occurred and suggested that progression of BM might not be due to limited drug exposure, but rather due to other molecular factors such as enhanced activity of Ras homolog family member A (RhoA). Nevertheless, a recent study by Veerman et al.,^21^ including patients also included in the current study, proposed that single nucleotide polymorphisms in the efflux transporters ABCB1 and ABCG2 were predictors of the development of CNS metastasis in patients with BM at start of osimertinib, implying that the intracranial osimertinib concentration does play a role in the development of BM and CNS metastasis.

This study has limitations. First, although all patients were enrolled in prospective biomarker studies, not all patients underwent appropriate plasma sampling according to predefined requirements; therefore, not all patients treated with osimertinib could be included in the study. Second, the sample size for the BM cohort was rather small and even though data from three hospitals were combined, the number of patients developing BM in both the BM and no/unknown BM cohorts was limited. Nevertheless, this is one of the largest studies combining osimertinib exposure and response data in patients with BM. Third, the low detection rate of BM in the no/unknown BM cohort could be due to lack of standard brain imaging. Although brain imaging is recommended in the current European Society of Medical Oncology guideline, it is not in the Dutch guidelines, and therefore not all patients underwent brain imaging at diagnosis of stage IV NSCLC or at start of osimertinib, and follow-up brain imaging in those patients was not always performed, unless there was clinical suspicion of BM. This lack of imaging results in potentially undetected asymptomatic development of BM in the no/unknown BM cohort results in possible underestimation of the cumulative incidence results for the no/unknown BM cohort; however, all patients with symptomatic BM were included. Fourth, we were not able to further distinguish the C_min,SS_ subgroups between patients who did receive cranial radiotherapy for BM and those who did not due to the already small number of patients in each C_min,SS_ subgroup. Cranial radiotherapy has been described to affect the BBB and thereby to influence the exposure of BM to TKIs.^33^ Fifth, osimertinib C_min,SS_ was estimated using a formula described by Wang et al.^25^ Drug elimination rate is a fixed factor in this formula, but it might fluctuate between individual patients due to slight interpatient variability in osimertinib clearance. Nevertheless, considering the population elimination long half-life of osimertinib of 44 hours on average, the impact of these aberrations on the estimated osimertinib C_min,SS_ is expected to be limited. Therefore, we expect minimal differences between the calculated C_min,SS_ and the actual C_min,SS_. Last, osimertinib has a high protein binding of 95%, which is predominantly dependent on serum albumin levels. In patients with low serum albumin levels, the unbound fraction of osimertinib could be increased. Nevertheless, low serum albumin levels are generally found in hospitalized patients and patients with malnutrition or cachexia (e.g., due to disease progression). Considering that the patients included in the current study received osimertinib in an outpatient setting and all osimertinib samples taken within three months before disease progression were excluded, it seems unlikely that low albumin levels substantially influenced our results. In conclusion, in the current study, we did not find a relation between osimertinib peripheral blood exposure (C_min,SS_), as a potential surrogate marker for intracranial osimertinib exposure, and the development or progression of BM in patients with advanced *EGFR*m*+* NSCLC receiving osimertinib. Future studies should be performed to identify the underlying causes of BM progression.

## CRediT Authorship Contribution Statement

**Judith L. Gulikers:** Conceptualization, Investigation, Methodology, Writing—original draft.

**G.D. Marijn Veerman:** Investigation, Writing—review and editing.

**Merel Jebbink:** Investigation, Writing—review and editing.

**Paul D. Kruithof:** Writing—review and editing.

**Christi M.J. Steendam:** Investigation, Writing—review and editing.

**René J. Boosman:** Investigation, Writing—review and editing.

**Ron H.J. Mathijssen:** Writing—review and editing.

**Vivianne C.G. Tjan-Heijnen:** Writing—review and editing.

**Johanna H.M. Driessen:** Methodology, Writing—review and editing.

**Safiye Dursun:** Writing—review and editing.

**Egbert F. Smit:** Writing—review and editing.

**Anne-Marie C. Dingemans:** Writing—review and editing.

**Robin M.J.M. van Geel:** Supervision, Writing—review and editing.

**Sander Croes:** Supervision, Writing—review and editing.

**Lizza E.L. Hendriks:** Supervision, Conceptualization, Writing—review and editing.

## Disclosure

Dr. Veerman reports receiving payment or honoraria for lectures from Eli Lilly (payed to the institute). Dr. Steendam reports receiving an unrestricted research grant from AstraZeneca (payed to the institute), payment or honoraria for lectures from Academic Pharmaceutical, supporting for attending meetings from Eli Lilly and Rochte and fullfilling unpaid roles at NVALT and CieBOD. Prof. dr. Mathijssen reports receiving grants of contracts for investigator initiated research from Astellas, Bayer, Boehringer-Ingelheim, Cristal Therapeutics, Novartis, Pamgene, Pfizer, Roche, Sanofi and Sevier (all payed to the institute). Prof. dr. Tjan-Heijnen reports receiving grants or contracts from Pfizer, Novartis, Eli Lilly, Gilead, Roche, AstraZeneca and Daiichi Sankyo (all payed to the institute), and receiving consulting fees from Eli Lilly, Novartis and AstraZeneca. Drs. Dursun reports receiving payment or honoraria a presentation at Novartis. Prof. dr. Smit reports receiving grants or contracts from AstraZeneca and Daiichi Sankyo (payed to the institute), receiving consulting fees from AstraZeneca, BMS, Boehringer-Ingelheim, Daiichi Sankyo, Sanofi, Eli Lilly, Roche Genentech, Merck, Novartis, Pfizer, Takeda and Taiho (all payed to the institute), participation on a data safety monitoring board or advisory board from DSI and Taiho and receiving payment of honoraria for lectures from Boehringer-Ingelheim. Prof. dr. Dingemans reports receiving grants or contracts from Amgen, Dutch Cancer Society and HANART (all payed to the institute), receiving consulting fees from AMGEN, Bayer, Boehringer-Ingelheim, Sanofy, Roche, Janssen and AstraZeneca (all payed to the institute), and receiving payment or honoraria for lectures from Janssen, Pfizer, AstraZeneca, Eli Lilly and Takeda (all payed to the institute), participation on a data and safety monitoring board or advisory board from Takeda and Roche and is unpaid char at EORTC LCG. Dr. van Geel reports receiving grants or contracts from Academic Alliance Fund (payed to the institute). Dr. Hendriks reports receiving grants or contracts from Roche Genentech, AstraZeneca, Boehringer-Ingelheim, Takeda, Merck, Pfizer and Novartis (all payed to the institute), receiving payment or honoraria for lectures from MSD, Eli Lilly, Sanofi, GSK and High5oncology (all payed to the institute), and personal payments from Benecke, Medtalks, Medimix, VJOncology, participation on a data safety monitoring board or advisory board from BMS, Eli Lilly, Roche Genetech, Pfizer, Takeda, MSD, Merck, Novartis, Boehringer-Ingelheim, Amgen, Janssen and Anhearts (all payed to the institute), and interview sessions funded by Roche Genentech, Bayer and Eli Lilly (all payed to the institute). All other authors declare no conflict of interest.
