# Supplementary material for: Osimertinib Plasma Trough Concentration in Relation to Brain Metastases Development in Patients With Advanced EGFR-Mutated NSCLC
Source: JTO Clin Res Rep. 2024 Feb 20;5(4):100656. doi: 10.1016/j.jtocrr.2024.100656 (PMC10973185; doi:10.1016/j.jtocrr.2024.100656)

**Appendix I: Calculation of osimertinib trough concentration**

$$C_{min,SS}=C\left( t \right)*\exp(k_{e}*\Delta T)$$

$$k_{e}= \frac{0.693}{t_{1/2}}$$

Where C_min,SS_ is the steady-state osimertinib trough concentration in ng/mL, C(t) is the measured osimertinib concentration in ng/mL, k_e_ is the elimination rate, t_1/2_ is the plasma population elimination half-life of osimertinib (44 hours) and ΔT is the difference between dosing interval and time post dose of the measured trough concentration (25).

**Appendix II: Baseline characteristics patients in the BM cohort**

|  | | C_min,SS,L_  (n = 12) (<159.3 ng/mL) | C_min,SS,M_  (n = 20)  (159.3 – 270.7 ng/mL) | C_min,SS,H_  (n = 17)  (> 270.7 ng/mL) |
| --- | --- | --- | --- | --- |
| Sex female, n (%) | | 4 (33.3) | 15 (75.0) | 16 (94.1) |
| Age, median + IQR in years | | 61 (13.5) | 63.5 (11.0) | 65 (7.0) |
| BMI, median + IQR in kg/m^2^ | | 24.0 (3.3) | 24.5 (5.4) | 24.8 (5.6) |
| Smoking status, n (%) | Never | 2 (16.7) | 10 (50.0) | 10 (58.8) |
|  | Current | 2 (16.7) | 1 (5.0) | 0 (0.0) |
|  | Former | 8 (66.7) | 9 (45.0) | 7 (41.2) |
| TP53 mutation, n (%) | | 7 (58.3) | 7 (35.0) | 8 (47.1) |
| Leptomeningeal metastasis n (%) | | 5 (41.7) | 6 (30.0) | 4 (23.5) |
| Line of treatment, n (%) | 1 | 9 (75.0) | 5 (25.0) | 2 (11.8) |
|  | 2 | 2 (16.7) | 11 (55.0) | 11 (64.7) |
|  | ≥ 3 | 1 (8.3) | 4 (20) | 4 (23.5) |
| Prior radiotherapy, n (%) | WBRT | 3 (25.0) | 1 (5.0) | 3 (17.7) |
|  | SRT | 2 (16.7) | 5 (25.0) | 1 (5.9) |
|  | No | 7 (58.3) | 14 (70.0) | 13 (76.5) |
| Prior TKI, n (%) | Erlotinib | 2 (66.7) | 6 (46.7) | 9 (60) |
|  | Afatinib | 1 (33.3) | 2 (6.7) | 1 (6.7) |
|  | Gefitinib | 0 (0.0) | 4 (26.7) | 1 (6.7) |
| Mutation, n (%) | Exon19del | 5 (41.7) | 12 (60.0) | 10 (58.8) |
|  | L858R | 5 (41.7) | 5 (25.0) | 3 (17.6) |
|  | Other | 2 (16.7) | 3 (15.0) | 1 (5.9) |
|  | Exon19del + L858R | 0 (0.0) | 0 (0.0) | 3 (17.6) |
| Abbreviations: n, number; IQR, Inter Quartile Range; BMI, Body Mass Index; TP53, Tumour protein p53; TKI, Tyrosine Kinase Inhibitor; BM, Brain metastases; WBRT, Whole Brain Radiotherapy; SRT, Stereotactic Radiotherapy; NA, Not Applicable. | | | | |

**Appendix III: Baseline characteristics patients in the no/unknown BM cohort**

|  | | C_min,SS,L_  (n = 31) (<159.3 ng/mL) | C_min,SS,M_  (n = 67)  (159.3 – 270.7 ng/mL) | C_min,SS,H_  (n = 26) (>270.7 ng/mL) |
| --- | --- | --- | --- | --- |
| Sex female, n (%) | | 18 (58.1) | 44 (65.7) | 23 (88.5) |
| Age, median + IQR in years | | 65 (18) | 65 (14) | 71.5 (17) |
| BMI, median + IQR in kg/m^2^ | | 24.2 (3.6) | 25.7 (4.5) | 24.8 (8.1) |
| Smoking status, n (%) | Never | 15 (48.4) | 40 (59.7) | 17 (65.4) |
|  | Current | 2 (6.5) | 1 (1.5) | 1 (3.9) |
|  | Former | 13 (41.9) | 26 (38.8) | 8 (30.8) |
|  | Unknown | 1 (3.2) | 0 | 0 |
| TP53 mutation, n (%) | | 16 (51.6) | 24 (35.8) | 9 (34.6) |
| Line of treatment, n (%) | 1 | 5 (16.1) | 19 (28.4) | 5 (19.2) |
|  | 2 | 19 (61.3) | 29 (43.3) | 15 (57.7) |
|  | ≥ 3 | 7 (22.6) | 19 (28.4) | 6 (23.1) |
| Prior TKI, n (%) | Erlotinib | 17 (65.4) | 31 (64.6) | 17 (81.0) |
|  | Afatinib | 2 (7.7) | 5 (10.4) | 1 (4.8) |
|  | Gefitinib | 8 (30.8) | 8 (16.7) | 2 (9.5) |
| Mutation, n (%) | Exon19del | 22 (71.0) | 41 (61.2) | 13 (50.0) |
|  | L858R | 7 (22.6) | 17 (25.4) | 9 (34.6) |
|  | Other | 1 (3.2) | 7 (10.4) | 3 (11.5) |
|  | Exon19del + L858R | 1 (3.2) | 2 (3.0) | 1 (3.8) |
| Abbreviations: n, number; IQR, Inter Quartile Range; BMI, Body Mass Index; TP53, Tumour protein p53; TKI, Tyrosine Kinase Inhibitor; BM, Brain metastases; WBRT, Whole Brain Radiotherapy; SRT, Stereotactic Radiotherapy; NA, Not Applicable. | | | | |

**Appendix** **IV. Cumulative incidence of BM progression in patients in the no/unknown BM cohort . C_min,SS,L_ (< 159.3 ng/mL) (blue); C_min,SS,M_ (159.3 – 270.7 ng/mL) (green); C_min,SS,H_ (>270.7 ng/mL) (red).**


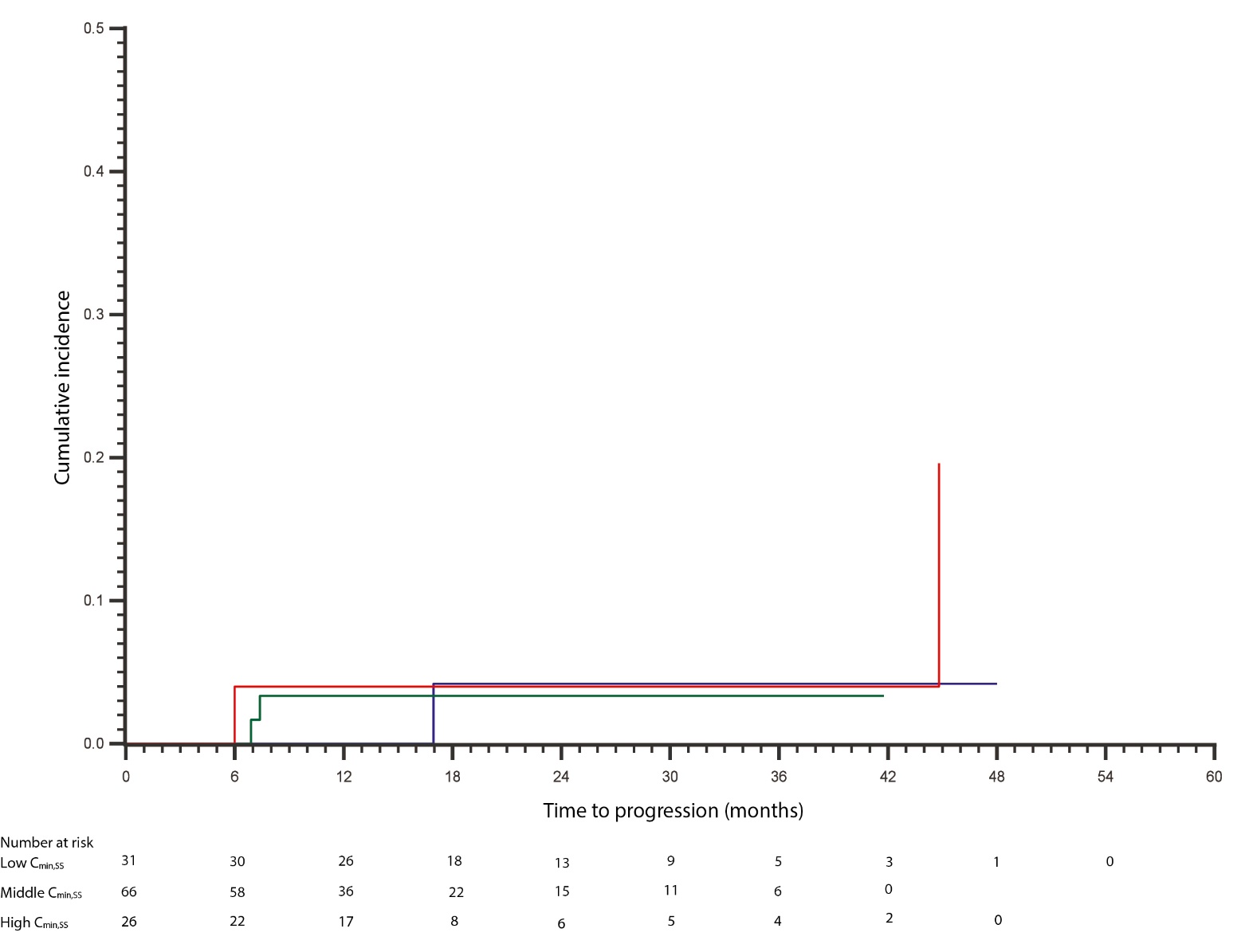


**Appendix V. Overall survival of patients in the BM cohort. C_min,SS,L_ (< 159.3 ng/mL) (blue); C_min,SS,M_ (159.3 – 270.7 ng/mL) (green); C_min,SS,H_ (>270.7 ng/mL) (red).**


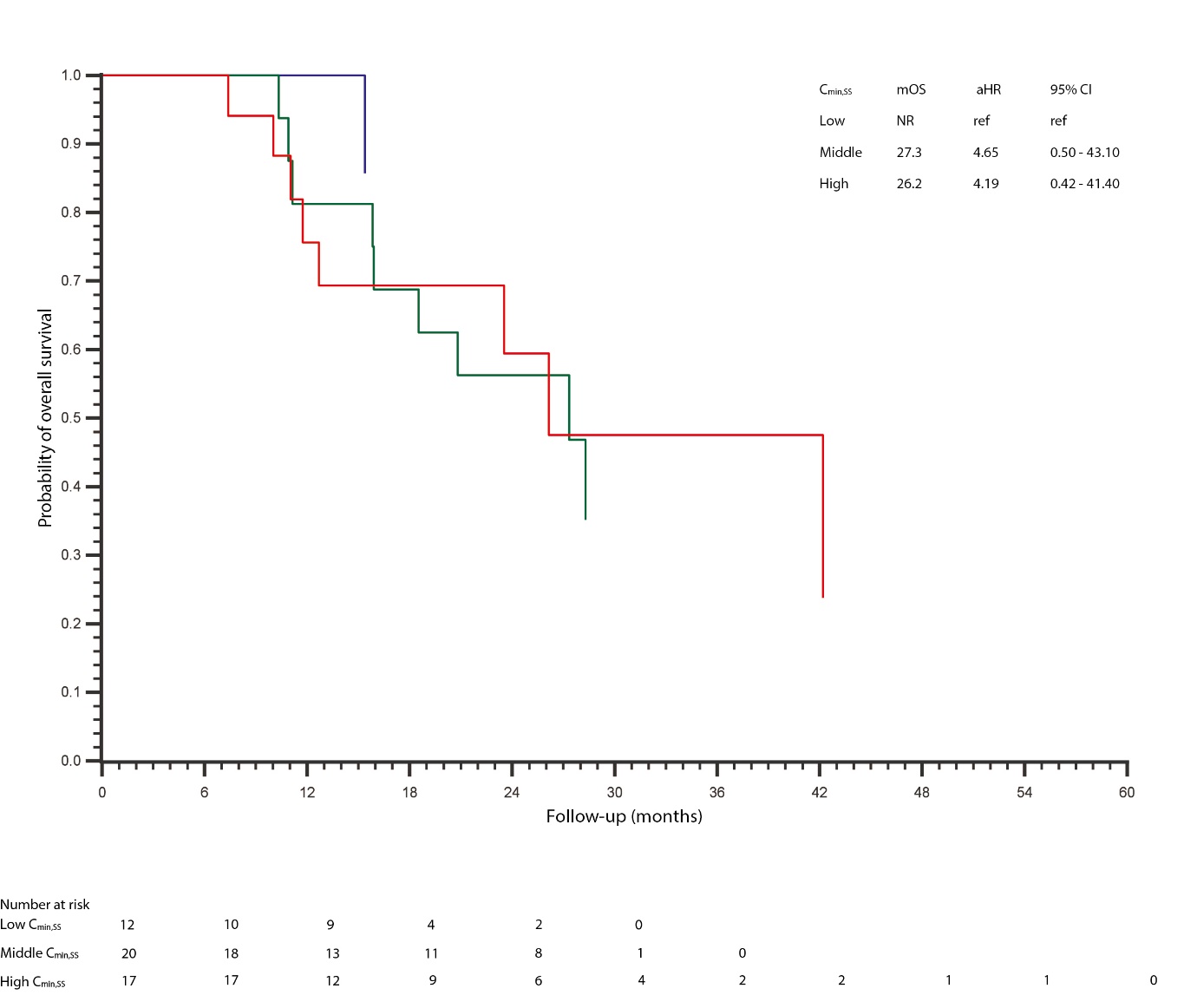


**Appendix VI. Overall survival of patients in the no/unknown BM cohort. C_min,SS,L_ (< 159.3 ng/mL) (blue); C_min,SS,M_ (159.3 – 270.7 ng/mL) (green); C_min,SS,H_ (>270.7 ng/mL) (red).**


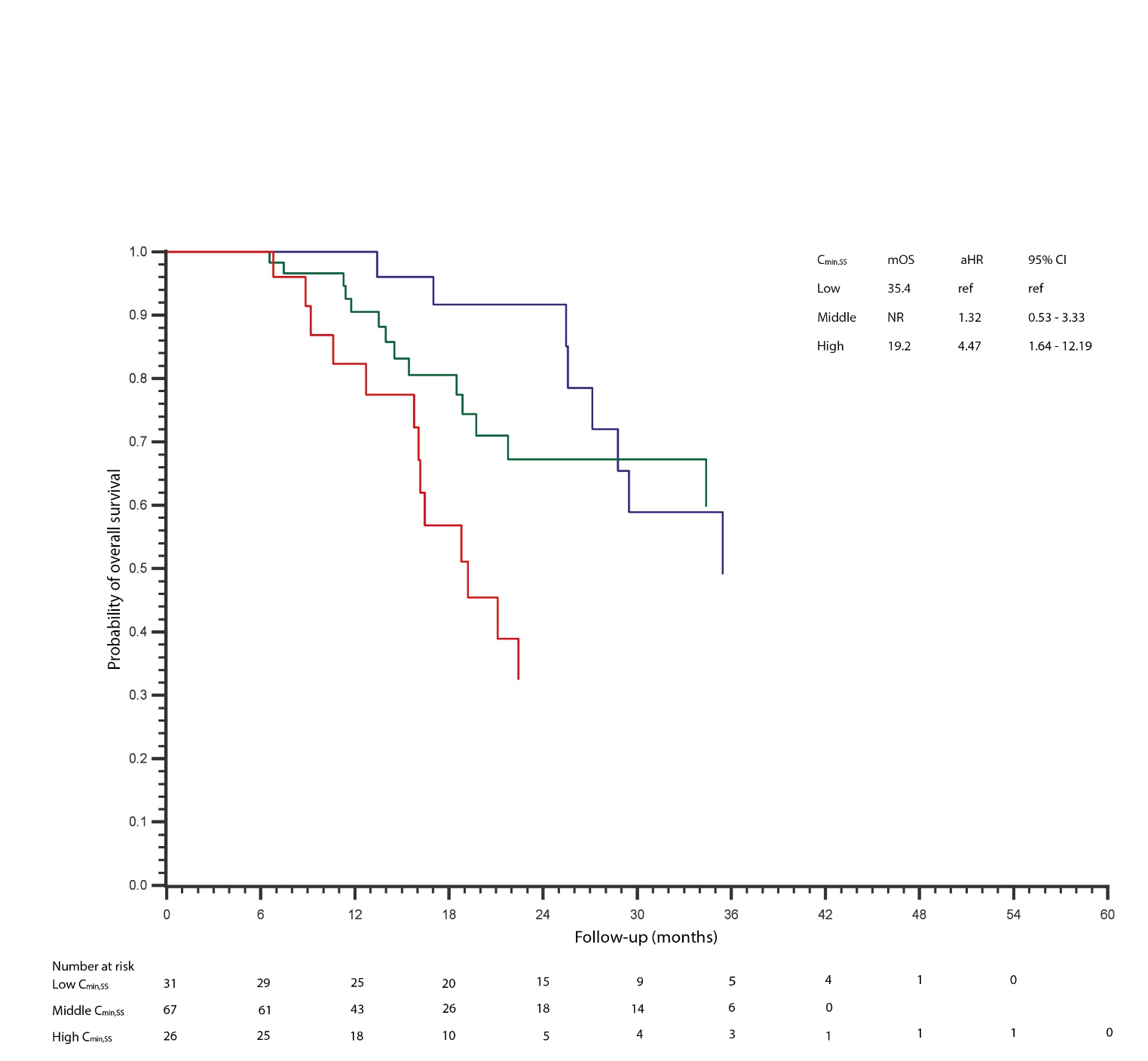

Supplement: Supplementary Appendix [file mmc1.docx]
